# A Retrospective Survey of the Abortion Outbreak Event Caused by Brucellosis at a Blue Fox Breeding Farm in Heilongjiang Province, China

**DOI:** 10.3389/fvets.2021.666254

**Published:** 2021-06-15

**Authors:** Yulong Zhou, Ye Meng, Yachao Ren, Zhiguo Liu, Zhenjun Li

**Affiliations:** ^1^College of Animal Science and Veterinary Medicine, Heilongjiang Bayi Agricultural University, Daqing, China; ^2^Department of Heilongjiang Key Laboratory for Animal Disease Control and Pharmaceutical Development, College of Veterinary Medicine, Northeast Agricultural University, Harbin, China; ^3^Pharmacy Department, Harbin Medical University-Daqing, Daqing, China; ^4^Chinese Center for Disease Control and Prevention, National Institute for Communicable Disease Control and Prevention, Beijing, China

**Keywords:** *Brucella melitensis*, abortion, reproductive, blue fox, goats (sheep) offal

## Abstract

Brucellosis is a common zoonosis in China, resulting in abortion in animals. Outbreaks of abortion in blue foxes caused by *Brucella* infection have rarely been reported. In the present study, 3–5 mL blood samples collected from the femoral veins of 10 abortuses of blue foxes were assessed by RBPT (Rose Bengal plate test) and SAT (serum tube agglutination test) to preliminarily investigate the source of infection for the clustering of abortion events at a blue fox farm in Heilongjiang Province. Screening experiments showed that all 10 blood samples were positive in the RBPT, while only eight blood samples out of the 10 were positive in the SAT. Subsequently, 10 tissue samples (spleen, lungs, stomach contents, and afterbirth) from the same 10 foxes were assessed using AMOS (acronym for *B. abortus, melitensis, ovis*, and *suis*)-PCR (polymerase chain reaction), and sequencing analysis was performed on amplification products to verify the results of the serology survey. Results showed a spectral band of ~731 bp in these samples. BLAST showed sequences of AMOS-PCR products in this study to be 100% similar (*E* = 0.0) to sequences in *B. melitensis* strain from GenBank. These data preliminarily indicated that the blue fox's outbreak of abortion events was caused by brucellosis via the *B. melitensis* strain. Then 726 serum samples were tested by RBPT and SAT to determine the prevalence of brucellosis on the farm. A comprehensive epidemiological and reproductive status survey of the infected blue fox population was performed. The seropositive rate was found to be 67.90% (493/726) by RBPT and 41.32% (300/726) by SAT. The technicians had stopped feeding the foxes with chicken carcasses and instead fed them raw ground sheep organs (lungs, tracheae, placentae, and dead sheep fetuses) infected by *B. meliteneis* strains, and that this change in diet caused the outbreak of abortion events. The high abortion rate (55%) and low cub survival rate (65%) were the most distinctive features of the outbreak; these factors led to severe economic losses. Feeding cooked sheep/goat offal and strict breeding management is necessary for disease prevention.

## Introduction

Brucellosis is a widespread zoonotic disease that is caused by bacteria and is categorized as a bacterial human disease (1). The World Organization for Animal Health (OIE) lists brucellosis as a multi-animal comorbidity (2), and brucellosis is a second-category animal infectious disease in China (3). The disease mainly affects the reproductive systems of animals (4, 5). Although 12 *Brucella* species have been identified, *B. melitensis, B. abortus*, and *B. suis* are the most common pathogens occurring in human and animal infections (6). Among domestic animals, cattle, sheep, and pigs are infected most frequently, and the disease can be transmitted to bison, elk, wild boars, foxes, hares, African buffalo, and reindeer (7). Brucellosis has caused huge economic losses in the animal husbandry and economic animal breeding industries worldwide (8, 9). The highest and lowest prevalence rates of brucellosis among different fox species were found in red fox (*Vulpes vulpes*) (100%) and hoary fox (*Lycalopex vetulus*) (9%), respectively (10). A study showed *Gardnerella vaginalis* to be the main pathogen that causes miscarriage in foxes in China; the seropositivity rate range of fox population in China is 0.9–21.9%, and in some farms it exceeds 75% (11). Canine distemper virus, pseudorabies virus, and *Staphylococcus aureus* are common pathogenic agents in the fox population (12), but there is no report of fox abortion caused by *Brucella* spp. Moreover, the incidence of brucellosis in China has continued to rise in recent years. Heilongjiang Province was designated a Type I brucellosis severe epidemic region due to the ongoing high incidence rate of animal brucellosis (13, 14). The animal husbandry industry is a main economic pillar of this province, and fox and raccoon breeding are the main sources of income for many farmers in this region. In March 2017, an outbreak of abortion of unknown origin occurred at a blue fox breeding farm in Heilongjiang Province, resulting in a high rate of abortion in pregnant blue foxes and causing serious economic losses. At present, serological techniques remains the mainstay for brucellosis diagnosis (15). These include the Rose Bengal Plate Test (RBPT), serum agglutination test (SAT), and complement-fixation test (CFT) (16–18). However, CFT is a technically complex test, and it requires good laboratory facilities and well-trained personnel to perform it accurately and maintain its reagents (19). Moreover, identification of *Brucella* sp. by conventional tests involves considerable time, risk of human infection, and expert interpretation, whereas PCR is fast, safe, and easy to interpret (20, 21). Previous works described a *Brucella* PCR assay that can distinguish *Brucella abortus* (biovars 1, 2, and 4), *Brucella melitensis* (biovars 1, 2, and 3), *Brucella ovis*, and *Brucella suis* (biovar 1) from each other (22). In this study, RBPT, SAT, and AMOS (*B. abortus, B. melitensis, Brucella ovis*, and *Brucella suis*)—PCR were used to determine the cause of the outbreak of abortions at a blue fox farm. Our investigation will provide important data for technical guidance in the prevention of blue fox brucellosis as well as promote better management of blue foxes in Heilongjiang province, China.

## Methods

### Serological Testing

Blood samples were collected from the femoral vein, 3–5 mL per blue fox. A total of 10 serum samples (HBF001–010) were collected from 10 female foxes that had miscarried during 15–20 days in April 2017, and 726 serum samples [65 male foxes, 34 male cub foxes (<1 year old), 564 female foxes, and 61 female cub foxes (<1 year old)] from the blue fox farm were collected in October 2017 to implement the epidemiological survey. Both the Rose Bengal plate test (RBPT) and the Serum Agglutination Test (SAT) were performed according to standard serological procedures (23). RBPT and SAT were used to diagnose human brucellosis (23). RBPT antigen (production batch number: 201701) and SAT antigen (production batch number: 201702) were purchased from Qingdao Yibang Bioengineering Co., Ltd.; brucellosis positive control serum (production batch number: 201702) and negative control serum (production batch number: 201701) were purchased from China Veterinary Drug Supervision Institute. Sperm samples collected from male foxes were preliminarily screened for quality by microscopic examination. Some medicines, including oxytetracycline, astragalus polysaccharides, Vitamin E, and other herbs, were used to treat the blue foxes.

### AMOS-PCR

The 10 tissue samples (liver, spleen, lungs, stomach contents, and afterbirth) from the same 10 aborted blue fox fetuses were collected following biosafety regulations. DNA of all samples was extracted using a Qiagen genome DNA prepare kit (Qiagen, Germany) according to the manufacturer's instructions. Subsequently, AMOS-PCR was employed to discriminate the species/biovar of *Brucella* strains. Amplification and detection procedures were as previously described (24). Briefly, the concentration of the four primer pairs was 25 μM/L, and primer A 1 μL, primer M 1.5 μL, primer O 1.5 μL, primer S 1 μL, primer IS711 2 μL, Taq DNA polymerase 1.25 U, and DNA template 2 μL. Finally, sterilized double distilled water was added to a final volume of 50 μL. Amplification parameters: 94°C pre-denaturation 5 min; 94°C 1 min, 60°C 1.5 min, 72°C 10 min, for 40 cycles; final extension at 72°C for 10 min. Five microliter products and 1 μL loading buffer were uploaded to agarose gels to determine the sizes of products. The target gene size was 498 bp for *B. abortus* (bv. 1, 2, and 4), 731 bp for *B. melitensis*, 976 bp for *B. ovis*, and 285 bp for *B. suis* (bv. 1). Then, 10 AMOS-PCR products were sequencing using M primer (F) and comparison was performed using the Basic Local Alignment Search Tool (BLAST).

### The Evaluation of Reproductive Performance in Female Blue Foxes

The breeding conditions, estrus rate, weak cub rate, abortion rate, disease occurrence, and medication use of the blue fox farm from 2017 to 2019 were investigated to determine the production performance impact of a female blue fox infected with *B. melitensis*.

## Results

### Serological Tests

In order to investigation the cause of the outbeak abortus event. First, ten samples from female foxes were collected and examined by RBPT and SAT. The RBPT results in all 10 serum samples from female foxes were positive. However, eight samples were positive for the SAT (titer 1:50, ++), while the two remaining samples were all suspect cases (titer 1:50, +) (Supplementary Table 1). A preliminary serological survey indicated that infection with *Brucella* spp. could be a cause of spontaneous abortion in blue foxes. Subsequently, for further survey the situation the infection in blue fax farming, a total of 726 serum samples were collected and detected by RBPT and SAT. The positive rate of the RBPT was 67.90% (493/726) (Table 1), and the positive rate of the SAT was 41.32% (300/726) (Table 1). The SAT titer in 125 samples was 1:25 + (Table 1). Finally, eight of the human staff of this far were screened for serum antibodies against *Brucella* infection in eight staff in this farming were performed, five staff members of the farm were diagnosed with brucellosis, while there were no brucellosis antibodies detected in the other three staff members. The obvious clinical symptoms (swollen testicles, bedridden, back pain, leg pain) were observed in five brucellosis patients. They frequently ground the raw internal organs of sheep/goat to feed the blue foxes.

**Table 1:** Brucellosis epidemic situation as detected by serological tests in 726 serum samples from blue fox breeding farm.

**Methods**	**Male**	**Male cub foxes**	**Female foxes**	**Female cub foxes**	**Total (%)**
RBPT (%)	61.58 (40/65)	11.76 (4/34)	78.55 (443/564)	8.20 (5/61)	67.90 (493/726)
SAT (%)	38.49 (25/65)	0	48.58 (274/564)	1.63 (1/61)	41.32 (300/726)

*RBPT, Rose Bengal plate test; SAT, serum tube agglutination test*.

### AMOS-PCR Amplification

**Figure 1:**
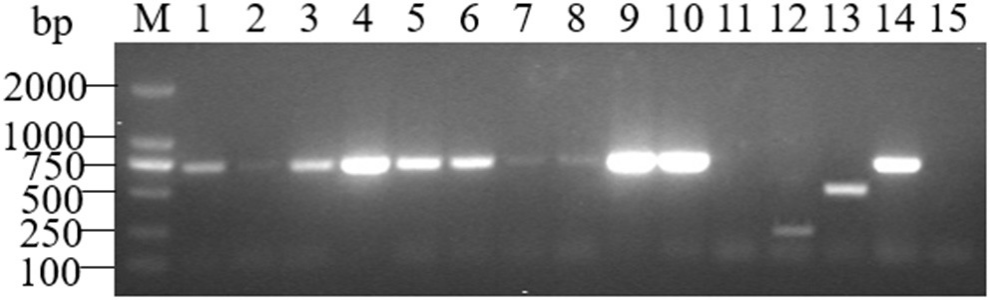
AMOS-PCR typing of the sample from three aborted fetuses of the blue fox. M, marker, DL2000 bp; lane 1–3, spleen, lungs and stomach contents samples from HBF001; lane 4–6, spleen, lungs and stomach contents samples from HBF002; lane 7–8, spleen and lungs samples from HBF003; lane 9–11, Fetal coats samples from three aborted fetus (HBF001-3); lane 12, *B. suis* S2; lane 13, *B. abortus* A19; lane 14, *B. melitensis* M5; lane 15. Negative control*, E. coli*.

The AMOS-PCR showed that the expected 731 bp size amplified result was observed in three positive controls (*B. melitensis* M5; 6. *B. abortus* A19, and *B. suis* S2), and there were no bands in the negative control *E. coli* strain. Moreover, an expected 731 bp band was detected among four different tissue types in the samples from aborted fetuses, including spleen, lung, stomach contents, and fetal coats, consistent with the target gene fragment of *B. melitensis* strains (Figure 1). PCR product sequencing showed that sequences ~700 bp in size were obtained from all 10 samples. Further BLAST showed that these sequences were 100% similar (*E* = 0.0, sort by percent identity as 100%) to sequences of *B. melitensis* strain hosted in GenBank (Supplementary Figure 1). This result further verified the results from serological tests as well as confirming that *B. melitensis* was the pathogen involved in the blue fox cluster of abortion events.

### The Epidemiology Investigation

The farm began breeding blue foxes in 2014. In 2016, there were 2,000 female foxes and 110 male foxes, and the abortion rate was 5%. Blue foxes started mating in March 2017, and miscarriages occurred 10–40 days after pregnancy [in general, around 53 days (49–56) for the entire pregnancy]. Although the female fox's estrus rate was 85% (1,700/2,000) in that year, the miscarriage rate was 50% (850/1,700); weak cubs accounted for 5% (250/5,000), and the mortality rate of foxes reached 35% (1,750/5,000) (Table 2). After the brucellosis was diagnosed, oxytetracycline, astragalus polysaccharides, Vitamin E, and other herbs were used for treatment, but these had no effect. Therefore, only 18 brucellosis-positive female foxes were kept for breeding in 2018, and the remainder were eliminated. The investigation found that from August 2016 to November 2016, previously used chicken carcasses were replaced by raw ground sheep internal organs (lungs, tracheae, placentae, and dead fetuses) to feed breeding foxes, and clustering of female fox abortions occurred a few months later. After being infected, female blue foxes were without any obvious manifestations; however, reduced sperm counts and deformed sperm in male foxes were observed in microscopic examinations (unpublished).

**Table 2:** The reproduction profile of the blue fox in this farm during 2016–2018.

**Years**	**Female no**.	**Estrus rate (%)**	**Abortion rate (%)**	**Survival rate (%)**
2016	2,000	93.33	5	98
2017	2,000	85.00	50	65
2018	18	77.78	7.14	33.33

*2017 is the year the infection occurred*.

## Discussion

Brucellosis is one of the most important infectious causes of reproductive disorders in various species of animals (25). Various *Brucella* species are well-known causes of contagious abortion in cattle, sheep, goats, swine, and other animals (26). In the present study, both serological and AMOS-PCR methods confirmed that a *Brucella* spp. strain was the cause of the outbreak of abortion among blue foxes on this farm. Similarly, a previous study reported that brucellosis was found in a fox farm (27). Molecular tools can support the results from serological tests to avoid cross-reaction with other pathogens (28). AMOS-PCR results showed the presence of this special 731 bp band in many aborted fetuses' samples. Moreover, sequences from PCR products have 100% similarity to *B. melitensis* sequences from GenBank. These data indicate that the outbreak at the blue fox farm was causing by the *B. melitensis* infected. A similar study showed that *B. melitensis* biovar 3 was the main pathogen responsible for cow and sheep abortion in China, and that this variant posed a human health risk (29). The seroprevalence of brucellosis in sheep and goat flocks was higher in eastern China, with 7.00% positive rate, than in any other region (30). Heilongjiang Province is one of the severe animal brucellosis epidemic regions in northern China (30). Moreover, ~9% (56/621) of the samples from yaks were seropositive for *Brucella* tested via SAT at the Qinghai-Tibet Plateau, China (31). Similarly, the individual yak seroprevalence of brucellosis was 2.8% and herd level seroprevalence was 18.2% (32). Also, *Brucella* strains were isolated from the wildlife in China, such as blue sheep (*Pseudois nayaur*), yaks (*Bos mutus grunniens*), and Tibetan gazelle (*Procapra picticaudata*) (33). *B. melitensis* biovar 3 from the spleen of an Asian badger (*Meles leucurus*) showed a MLVA-16 genotype similar to that of isolates from local aborted sheep fetuses (34).

Our surveys showed that sporadic abortion events occurred in 5% of pregnancies on this farm during 2016. However, a >50% abortion rate was observed in 2017. The blue fox farm did not introduce new foxes during the period 2014–2017, and the breeding environment had not changed. The only changed factor was the feed for the blue foxes, where raw ground offal of sheep from the local slaughterhouse was used to feed the breeding foxes instead of chicken carcasses as used previously. Subsequently, an outbreak abortion event occurred during March and April in 2017. Moreover, serological screening showed that the seropositive rate of brucellosis in the fox breeding farm was 41.32% (300/726), being 38.49% (40/64) in male foxes and 48.58% (274/564) in female foxes. Moreover, five out of eight staff in this farm were diagnosed with brucellosis. This evidence indirectly showed that feeding the raw viscera of sheep infected with *Brucella* spp. were the main cause for the outbreak of abortion events on the blue fox breeding farm. Due to the high abortion rate (55%), low cub survival rate (65%), and human infections, this farm was closed at the beginning of 2019. The study showed that the highest-threat organs of ruminants are the lungs, and the trend analysis also highlighted the cattle intestine as a potentially high-threat organ (35). Moreover, our previous study reported that *B. melitensis* was obtained from dogs that were often fed with sheep offal (36). Moreover, hares have been considered as a possible source of *B. suis* biovar 2 outbreaks in domestic pigs via swill feeding with offal from hunted infected hares (37).

In order to identify the causative pathogen of blue fox abortion, we tried to isolate and cultivate *Gardnerella vaginalis* and other common abortion-related pathogens, but only a few *Staphylococcus* and *Streptococcus* strains were detected in abortion afterbirth. What we particularly regret is that our laboratory (Heilongjiang Bayi Agricultural University) did not meet the expected biosafety requirements necessary for bacteriological experiments, so *Brucella* strains isolation were not performed. Isolated *Brucella* from the (wild) red fox (*Vulpes vulpes*) (38, 39), gray fox (40), and tundra wolf (41) have been reported. Therefore, our conclusion is a reasonable explanation for this outbreak of abortion events. In addition, blue foxes infected by *Brucella* strains were without any obvious symptoms except the abortion after pregnancy at 10–40 days. This observation agrees with a previous report that *B. melitensis* in the adult ewe is generally asymptomatic and self-limiting within about 3 months. However, because the bacteria may enter and cause necrosis of the chorionic villi and fetal organs, abortion or stillbirths may occur (42, 43). Another study showed that brucellosis is essentially a disease of sexually mature animals, the preferred site being the reproductive tracts of males and females. If the animal is not pregnant, the infected animal may be without clinical symptoms and may have a negative serological reaction. However, if such an animal becomes pregnant, the production of the simple carbohydrate erythritol in the fetus and its membranes causes rapid multiplication of bacteria in the uterus, and this is likely to end in abortion (44). In this study, a 77.78% (14/18) estrus rate was recorded in blue foxes after infection by *B. melitensis*. In comparison with 2016, the estrus rate had declined; the abortion rate was 10 times higher than previously, and the survival rate of the pups dropped significantly. *B. melitensis* primarily affects the reproductive tracts of sheep and goats, and the infection is characterized by late abortion, stillbirth, a weakened fetus, and to a lesser extent orchitis and infection of the accessory sex glands and impaired fertility in males (45). The stillbirths and weakened fetuses in this case resulted in economic losses. The infected staff member often participated in the offal grinding, and thus the specific source of infection needs further investigation. *B. melitensis* infects mainly sheep and goats and other animals, resulting in an important zoonosis that has a significant effect on the husbandry economy and the public health of many developing countries.

Our study has several limitations. Due to restrictions by the limited lab facilities, the isolation and culture of *Brucella* from abortus samples were not carried out. Moreover, a tracing-back survey of the source of sheep (goats) offal is lacking. Animal offal samples have been collected from the local slaughterhouse for further bacteriological experiments, and genetic phylogenetic analysis will provide the available information to reveal the complete transmission chain of events.

## Conclusion

In the present study, we combined RBPT, SAT, and AMOS-PCR to investigate the cause of an abortion outbreak event in a blue fox farm in Heilongjiang province. Our experiments showed that blue foxes ingesting sheep offal infected with *B. melitensis* was the main cause of the outbreak. These data indirectly verified the severe animal brucellosis epidemic trend in this region, where *B. melitensis* infection was a spillover from the main host to the blue fox. These events pose a public health risk to people in the fur and catering industries and to workers in other breeding industries that provide animal feed. It is thus time to launch an animal brucellosis prevention program against the spread of *Brucella*.

## Data Availability Statement

All data generated or analyzed during this study are included in this published article and its Supplementary Information files.

## Ethics Statement

The animal study was reviewed and approved by Ethics Committee of the Heilongjiang Bayi Agricultural University. Written informed consent was obtained from the owners for the participation of their animals in this study.

## Author Contributions

YZ, YM, and YR collected the samples and performed the serology and AMOS-PCR amplifications. ZLiu performed data analysis and drafted the manuscript. YZ and ZLiu conducted epidemiological investigations. YZ and ZLi participated in the design of the study, critically reviewed the manuscript, and managed the project. All authors have read and approved the final version of the manuscript.

## Conflict of Interest

The authors declare that the research was conducted in the absence of any commercial or financial relationships that could be construed as a potential conflict of interest.

## Abbreviations

RBPT: Rose-Bengal plate test
SAT: serum tube agglutination test
AMOS-PCR: B. abortus-*melitensis*-*ovis*-*suis* polymerase chain reaction.
