# Supplementary material for: A Retrospective Survey of the Abortion Outbreak Event Caused by Brucellosis at a Blue Fox Breeding Farm in Heilongjiang Province, China
Source: Front Vet Sci. 2021 Jun 15;8:666254. doi: 10.3389/fvets.2021.666254 (PMC8239190; doi:10.3389/fvets.2021.666254)
Supplement: Supplementary file 1 [file Table_1.docx]

Table. S1 Preliminary screening of brucellosis was performed by serological testing in ten blood samples from abortus female blue foxes.

| Samples No. | RBPT | SAT | Dilution gradient | | | | | | |
| --- | --- | --- | --- | --- | --- | --- | --- | --- | --- |
|  |  |  | 1:12.5 | 1:25 | 1:50 | 1:100 | 1:200 | 1:400 | 1:800 |
| HBF001 | ＋ | ＋ | ＋＋＋＋ | ＋＋＋＋ | ＋＋＋＋ | ＋＋＋＋ | ＋＋＋ | － | － |
| HBF002 | ＋ | ± | ＋＋＋＋ | ＋＋＋ | ＋ | － | － | － | － |
| HBF003 | ＋ | ＋ | ＋＋＋＋ | ＋＋＋＋ | ＋＋＋ | － | － | － | － |
| HBF004 | ＋ | ＋ | ＋＋＋＋ | ＋＋＋＋ | ＋＋ | － | － | － | － |
| HBF005 | ＋ | ＋ | ＋＋＋＋ | ＋＋＋＋ | ＋＋＋＋ | ＋ | － | － | － |
| HBF006 | ＋ | ＋ | ＋＋＋＋ | ＋＋＋＋ | ＋＋＋＋ | ＋＋＋＋ | ＋＋ | － | － |
| HBF007 | ＋ | ＋ | ＋＋＋＋ | ＋＋＋＋ | ＋＋＋＋ | ＋＋ | － | － | － |
| HBF008 | ＋ | ± | ＋＋＋＋ | ＋＋＋＋ | ＋ | － | － | － | － |
| HBF009 | ＋ | ＋ | ＋＋＋＋ | ＋＋＋＋ | ＋＋＋＋ | ＋＋＋＋ | ＋＋＋＋ | ＋＋ | － |
| HBF010 | ＋ | ＋ | ＋＋＋＋ | ＋＋＋＋ | ＋＋ | － | － | － | － |
| PC | ＋ | ＋ | ＋＋＋＋ | ＋＋＋＋ | ＋＋＋＋ | ＋＋＋＋ | ＋＋＋ | ＋＋ | － |
| NC | － | － | － | － | － | － | － | － | － |
| AC | / | － | / | / | / | / | / | / | / |

PC: positive control; NC: negative control; AC: antigen control; RBPT: Rose Bengal plate test; SAT: Serum Agglutination Test.

+: positive; ±: suspect; －: negative; /: no reaction.
