# Supplementary figures and images for: A Retrospective Survey of the Abortion Outbreak Event Caused by Brucellosis at a Blue Fox Breeding Farm in Heilongjiang Province, China
Source: Front Vet Sci. 2021 Jun 15;8:666254. doi: 10.3389/fvets.2021.666254 (PMC8239190; doi:10.3389/fvets.2021.666254)

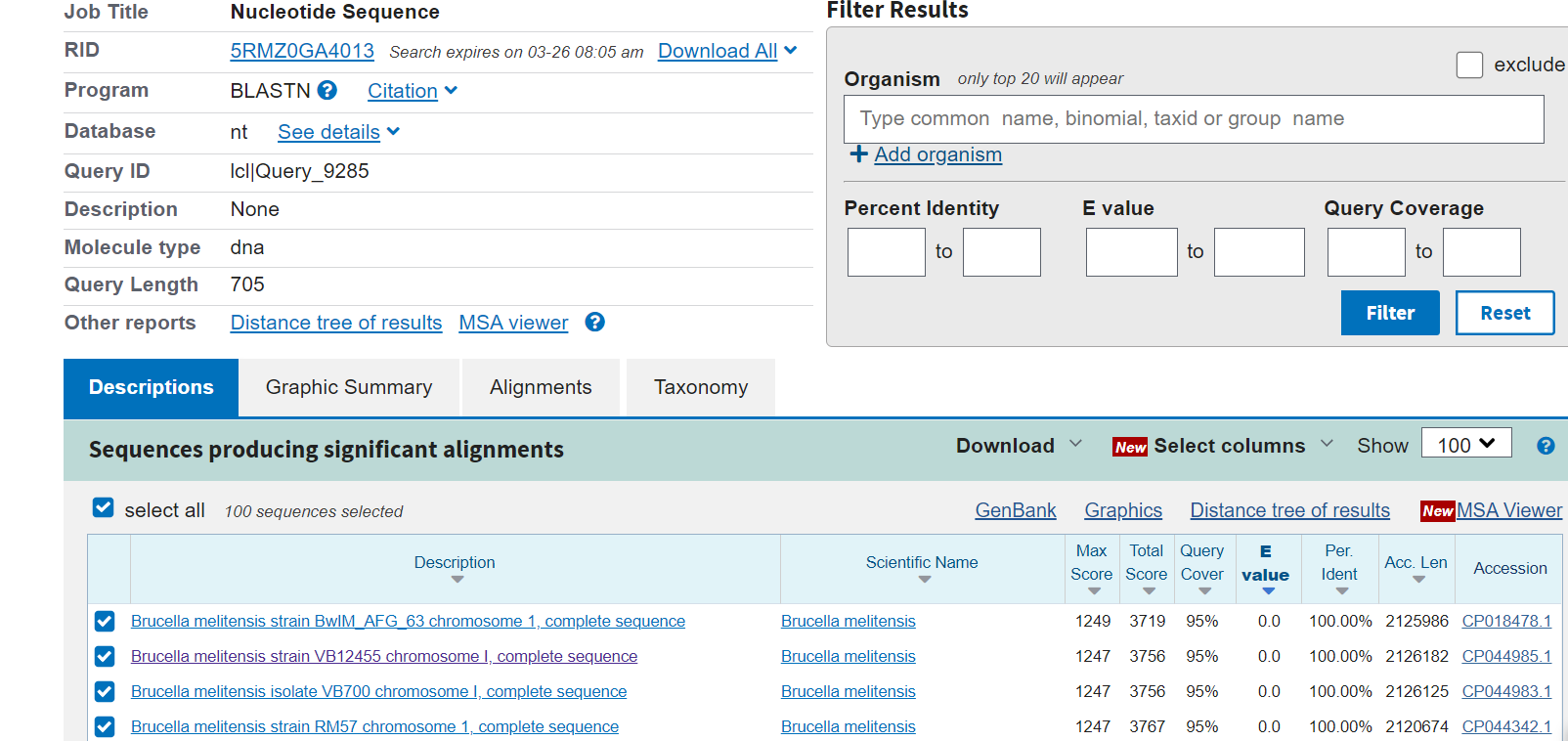

Supplement: Supplementary file 2 [file Image_1.TIFF]
